# Inhibition of α_v_β_3_ integrin induces loss of cell directionality of oral squamous carcinoma cells (OSCC)

**DOI:** 10.1371/journal.pone.0176226

**Published:** 2017-04-24

**Authors:** Cyntia F. Montenegro, Bruna C. Casali, Rafael L. B. Lino, Bianca C. Pachane, Patty K. Santos, Alan R. Horwitz, Heloisa S. Selistre-de-Araujo, Marcelo L. Lamers

**Affiliations:** 1 Department of Physiological Sciences, Center of Biological and Health Science, Federal University of São Carlos, Rod. Washington Luis, São Carlos, São Paulo, Brazil, CEP; 2 Department of Cell Biology, University of Virginia, School of Medicine, Charlottesville, Virginia, United States of America; 3 Department of Morphological Sciences, Institute of Basic Health Sciences, Federal University of Rio Grande do Sul, Rua Sarmento Leite, Porto Alegre, RS, Brazil, CEP; Thomas Jefferson University, UNITED STATES

## Abstract

The connective tissue formed by extracellular matrix (ECM) rich in fibronectin and collagen consists a barrier that cancer cells have to overpass to reach blood vessels and then a metastatic site. Cell adhesion to fibronectin is mediated by α_v_β_3_ and α_5_β_1_ integrins through an RGD motif present in this ECM protein, thus making these receptors key targets for cell migration studies. Here we investigated the effect of an RGD disintegrin, Dis*Ba*-01, on the migration of human fibroblasts (BJ) and oral squamous cancer cells (OSCC, SCC25) on a fibronectin-rich environment. Time-lapse images were acquired on fibronectin-coated glass-bottomed dishes. Migration speed and directionality analysis indicated that OSCC cells, but not fibroblasts, showed significant decrease in both parameters in the presence of Dis*Ba*-01 (1μM and 2μM). Integrin expression levels of the α_5_, α_v_ and β_3_ subunits were similar in both cell lines, while β1 subunit is present in lower levels on the cancer cells. Next, we examined whether the effects of Dis*Ba*-01 were related to changes in adhesion properties by using paxillin immunostaining and total internal reflection fluorescence TIRF microscopy. OSCCs in the presence of Dis*Ba*-01 showed increased adhesion sizes and number of maturing adhesion. The same parameters were analyzed usingβ3-GFP overexpressing cells and showed that β3 overexpression restored cell migration velocity and the number of maturing adhesion that were altered by Dis*Ba*-01. Surface plasmon resonance analysis showed that Dis*Ba*-01 has 100x higher affinity for α_v_β_3_ integrin than forα5β1 integrin. In conclusion, our results suggest that the α_v_β_3_ integrin is the main receptor involved in cell directionality and its blockage may be an interesting alternative against metastasis.

## Introduction

Oral squamous cell carcinoma (OSCC) is responsible for 90% of all oral cancers occurring at different sites of the oral mucosa [1, 2]. The exposure to chemical, physical or biological agents with mutagenic and carcinogenic properties leads mutated keratinocytes to proliferate and invade the connective tissue. OSCCs responsiveness to chemotherapy is low likely due to the heterogeneity of tumor cell population; the high local recurrence rate of this disease results in a 5 year survival rate of only 40–50% [2, 3].

Tumor cell migration is pivotal step in invasion and metastases. The change from a non-migratory to a migratory phenotype results in detachment from the primary tumor, invasion into the surrounding tissues, and entry into blood and lymph vessels [4, 5]. Throughout this process, epithelial-derived tumor cells need to adapt to the changing environment, reflecting the change from a laminin-enriched basal membrane to a connective tissue rich in fibronectin and collagen. Previous studies showed that more aggressive OSCCs correlate with increased presence of fibronectin throughout the tissue, particularly at the invasion front [6, 7], which might result in a fast single-cell migration mode [8].

Cell migration is a complex and cyclic process mediated by adhesion molecules such as the integrin receptors [9–11]. They are composed of two different subunits, α and β which may assemble into 24 differently combined heterodimers with individual specificities to ECM proteins. Although some integrins share affinity to the same ligands, they may trigger different signaling pathways [12–14] such as the integrins α_5_β_1_ and α_v_β_3_ that bind to fibronectin through a RGD (arginine-glycine-aspartic acid) motif but differentially regulates adhesion maturation [15, 16]. Therefore, the use of peptides that bind to integrins could be considered a strategy to impair cell motility and decrease the possibility of metastasis.

Therefore, the blockage of integrin activity affects several adhesion dependent processes as migration, proliferation, apoptosis, among other processes [17–21]. For instance, disintegrins, a family of low molecular weight, cysteine-rich peptides discovered in snake venoms were found to cause platelet aggregation inhibition, by binding to the integrin α_IIb_β_3_ [22, 23]. Dis*Ba*-01 (disintegrin of *Bothrops alternatus*) is a recombinant RGD containing disintegrin that inhibits cell migration [24], *in vivo* angiogenesis [25], pulmonary metastasis [26] and is able to decrease the expression of VEGF receptors in endothelial cells [27]. Since ECM components are able to modify the adhesion properties of tumor cells and elicit a migratory behavior [28], we hypothesized that the selective blockage of integrin receptors by an RGD disintegrin might impair the migratory process of OSCC cells on a fibronectin enriched environment.

In this study, we show that Dis*Ba*-01 decreased migration speed, directionality and changed the migration mode of a highly invasive OSCC cell line from single to collective cell migration but had no effect on fibroblasts. This selective effect on epithelial-derived tumor cells was accompanied by a modulation of adhesion dynamics and was rescued by the overexpression of β3 integrin. Together, these data strongly suggest thatαvβ3 integrin is the major receptor involved in cell speed and directionality of OSCCs and it can be a critical target for therapy against metastasis.

## Material and methods

### Dis*Ba*-01 expression and purification

Dis*Ba*-01 is a recombinant disintegrin produced from a cDNA venom gland library of *B*. *alternatus*, since the native disintegrin could not be purified from the venom due to low yields in protein preparations [26]. The coding region corresponds to an RGD containing motif disintegrin of 78 amino acids residues (GenBank accession AY259516). Expression and purification of the recombinant His-tag protein were performed as previously described [26]. Molecular modeling and adhesion assays suggested that the fusion His-tag peptide was not involved on integrin binding and therefore its proteolytic removal would not be needed [26–27].

### Cell culture and transfection

SCC25 cells (ATCC1 CRL-1628™) were grown in DMEM/F12 with 15mM HEPES and 0.5mM sodium pyruvate (Gibco), FBS 10% and hydrocortisone (400ng/ml, Sigma), while BJ cells (ATCC® CRL2522™) in DMEM Glutamax with 1% non-essential amino acids (NEAA) and 10% fetal bovine serum. Cal27 (ATCC1 CRL-2095™) were cultivated in DMEM high glucose (Gibco) supplemented with 10% Fetal Bovine Serum (FBS). All cells were maintained in incubator at 37°C and 5% CO_2_. SCC25 cell was considered a highly aggressive oral squamous cell carcinoma (OSCC) cell line due to its low E-cadherin content when compared to Cal27, an OSCC cell line considered to be minimally invasive [28]. For Total Internal Reflectance Fluorescence (TIRF) microscopy, SCC25 were transfected using TransIT-2020 (Mirus) 24hs before the experiment with 0.5μg of paxillin-GFP plasmid [29]. For β3 subunit overexpression studies, cells were transfected with 0.5μg β3-GFP and 0.5μg paxillin-mko.

#### Migration and adhesions dynamics assays

Both assays used 3cm glass-bottomed dishes containing fibronectin (2μg/mL) and/or Dis*Ba*-01 (1 and 2μM) as a substrate or diluted in the media. Cells were plated with serum free media (CCM1, Thermo Fisher Scientific) and left in the incubator at 37°C for 1 hour (phase microscopy) or 20 min (TIRF microscopy) before imaging. At least 3 different experiments were performed for each group.

For time-lapse phase microscopy, images were acquired from either: BJ (human fibroblasts) and SCC25 (OSCC) over 8 hours with a 10 minute interval, at 37°C using a Nikon TE300 microscope (10x 0.25 NA CFI Achro DL106 Nikon objective), with a charge coupled device camera (Orca II, Hamamatsu Photonics, Iwata-City, Japan) using Metamorph software (Molecular Devices), as previously described [30]. Image J software in manual tracking was used to analyze migration parameters. Migration speed was determined by the ratio between the total distance and duration of cell migration; while directionality was determined by X and Y coordinates normalized to a “zero” starting point.

For time-lapse movies on TIRF microscopy, only the SCC25 cell was used. Images were captured every 3 seconds over 20 minutes using an Olympus IX70 inverted microscope (63x 1.45 NA oil Olympus PlanAplo 660 TIRFM objective), fitted with a Ludl modular automation controller (Ludl Electronic Products), a charge-couple device camera (RetigaExi, Qimaging), and Metamorph software. GFP was excited using the 488nm laser line of an Argon laser (Melles Griot), a dichroic mirror (HQ485/30) and an emission filter (HQ525/50). To estimate the rate of adhesion maturation, nascent adhesions were identified as the adhesions formed at the border of the cell membrane during protrusion and mature adhesions were identified as those that persisted through membrane protrusion, growing in size and elongating.

Additionally, invasion assays were made using 24-well plate Matrigel™ invasion chambers (Corning). DMEM/F12 medium with 5% FBS was pipetted into the wells, except on the negative control where serum-free F12 medium was used. SCC-25 cells (1 x 10^5^ cells) were treated with 1000 nM or 2000 nM Dis*Ba*-01 on serum-free F12 medium for 30 minutes. After that, the membrane-containing chamber was added to the well and treated or not-treated cells were applied. The invasion occurred for 48 hours at 37°C. The chamber membrane was fixed in 4% paraformaldehyde and cell nucleus was stained using DAPI. Membranes were assembled in slides using Vectashield^®^ mounting media (Vector Laboratories) and cell count was performed on automated fluorescence microscope system, ImageXpress Micro (Molecular Devices).

#### Western blots

Cells were trypsinized, washed and lysed in RIPA Buffer (25mMTris-HCL pH 7.6, 150mM NaCl, 1% NP-40, 1% sodium deoxycholate, 0.1% SDS) with protease (P8340, Sigma Aldrich) and phosphate cocktails (P8340, Sigma Aldrich). The lysates were separated by 4–20% precast polyacrylamide gel (BioRad) and proteins transferred to PVDF membranes (BioRad), blocked for 1 hour with 5% defatted milk in a PBS/0.5% Tween 20 at room temperature. Samples were immunoassayed for α_v_, β_3_, α_5_, β_1_, E-cadherin, N-cadherin and α-Tubulin (Cell Signaling, USA) using Pierce ECL Western Blotting Substrate (Thermo Scientific).

#### Immunofluorescence

Cells were incubated with Dis*Ba*-01 for 3 hours on fibronectin (2μg/ml) covered glass coverslips on CCM1 media. Then, cells were washed with PBS, fixed with formaldehyde 4%, and permeabilized using TritonX-100 (0.3%). Nonspecific binding sites were blocked with normal goat serum, and the cells incubated overnight with anti-paxilin antibody (BD Bioscience). After washing, cells were incubated with secondary antibodies against mouse bound to Alexa488 dye (Molecular Probes, Oregon, USA) and the actin filaments were marked with phalloidin conjugated to rhodamine (Molecular Probes, Oregon, USA). Images were obtained on a confocal microscope (Olympus FluoView 1000, Tokyo, Japan) with the 63x objective (UPlanSApo x63, 1.20 NA, oil immersion objective). Alexa488 was excited via the 488nm line of an Ar laser (Melles Griot, Albuquerque, NM), and rhodamine via the 543nm line of a Helium-Neon laser (Melles Griot, Albuquerque, NM). Images were acquired using FluoView software (Olympus, Tokyo, Japan). The Z-stacks were acquired from cells using 3 0.1μm step size slices (0.3μm of the cell) that were merged using the ImageJ software tool “Z-stack/sum”.

#### Surface plasmon resonance (SPR)

The affinity of Dis*Ba*-01 to immobilized integrins was measured using SPR (BIAcore T200; GE Healthcare, Little Chalfont, UK). α_5_β_1_ and α_V_β_3_ integrins (R&D, Minneapolis, MN, USA) were covalently attached via amine coupling (pH Scouting) to sensor chip CM5(GE Healthcare, Little Chalfont, UK). The bound ligand was then perfused in buffer containing 0.01 M Hepes, pH 7.4, 0.15 M NaCl, and 0.005 (v/v) Surfactant P20 (HBS–EP) at 25°C and flow rate of 30 μL/min. Association and dissociation constant rates were calculated by curve fitting, using BIA evaluation 1.1 software, assuming that Dis*Ba*-01 is monomeric with a MR of 12,000 Da. After testing the sensor chip in a pH gradient (pH Scouting), integrins α_5_β_1_ (pH 4.5) and α_V_β_3_ (pH 4.0) were immobilized onto the sensor chip to get a 4,325.2 RU (Resonance Unit) and a 6,677.1 RU, respectively. Chip regeneration was done by injection of Gly HCl 2M, pH 2.0 for 10s. Dis*Ba*-01 (0.1–100 μM) was injected at increasing concentrations onto the sensor chip with both immobilized integrins and the association constant between Dis*Ba*–01 and the integrins was calculated via GraFit 5.0 software.

### Statistical analysis

One way analysis of variance (ANOVA) was used followed by Tukey´s post test and student t-test for the TIRF microscopy assays.

## Results

### Dis*Ba*-01 selectively affects OSCC migration

**Fig 1:**
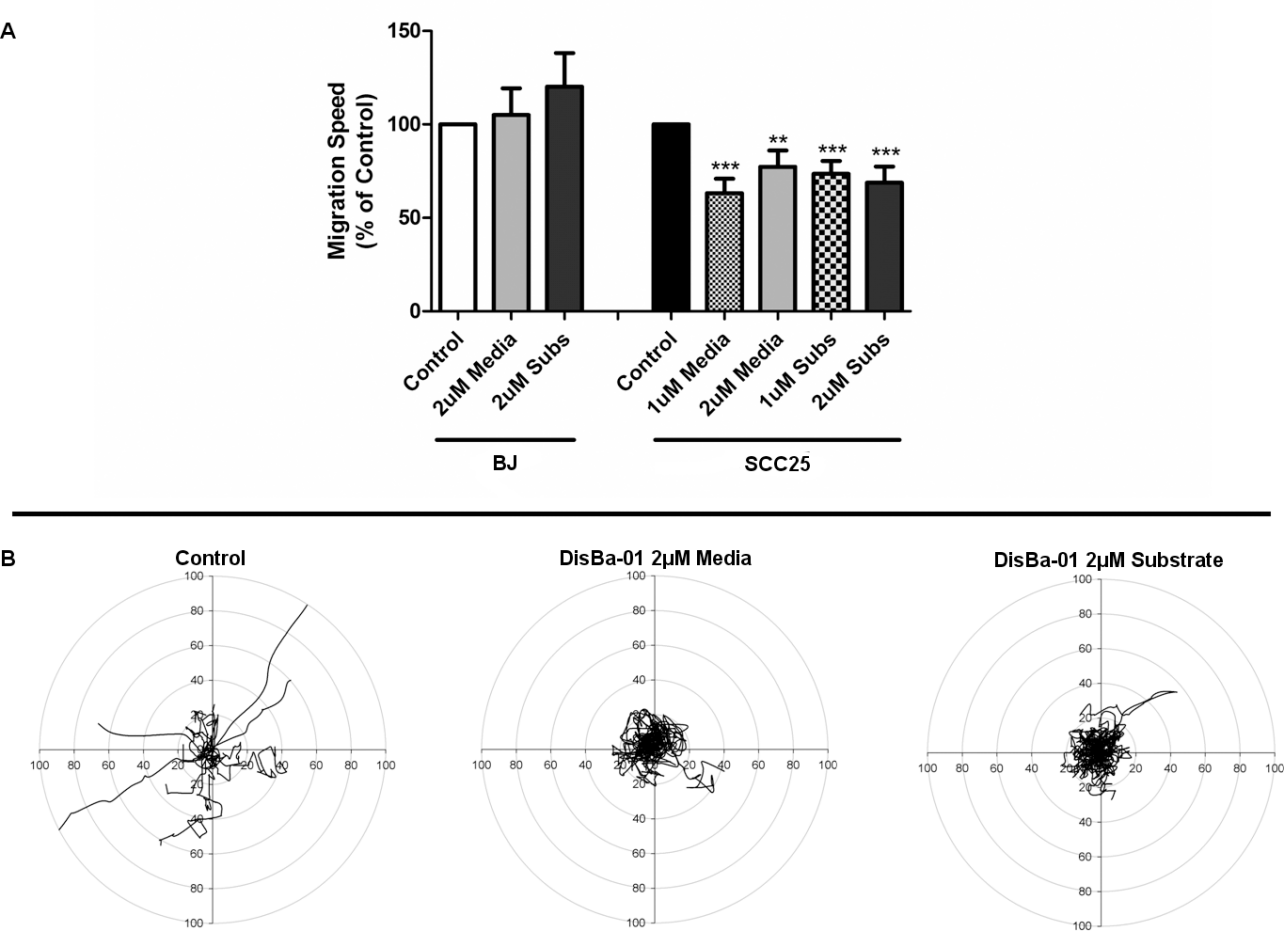
Dis*Ba*-01 inhibits the migration of OSCC cell line. (A) Dis*Ba*-01 significantly inhibited the migration speed of Oral Squamous Cell Carcinoma cells (SCC25) cells but not in fibroblasts (BJ) on both conditions tested (Media = DisBa-01 in the media; Subs = DisBa-01 in the substrate). Results were calculated as % of control. Statistical analysis was performed using ANOVA (one-way) followed by Tukey’s post-test, p<0.0001. (B) Migration tracks of SCC25 cells treated with Dis*Ba*-01 in the concentrations of 2μM indicates a loss of directionality when compared to the control tracks. Each individual line represents a cell path translated to a common origin.

As shown in our previous work, Dis*Ba*-01 inhibits cancer cell and fibroblast migration using a transwell migration assay [24]. Therefore, to investigate its potential effect on cell migration in a fibronectin rich environment we used two human cell lines that exhibit single cell migration: fibroblasts (BJ) and a highly invasive/low differentiated oral squamous carcinoma cell line (SCC25).Time-lapse movies (8h) were performed on both cell lines plated on glass-bottomed dishes coated with fibronectin (2μg/ml) in the presence/absence of Dis*Ba*-01 (1 or 2μM) in the media or in the substrate. Individual migratory cells were tracked and analyzed for migration speed and directionality (total net translocation from the origin point). The fibroblasts (n = 3 experiments) were not detectably affected by Dis*Ba*-01, even at a high concentrations (2μM) when compared with control cells (Fig 1A, S1 Movie). However, the OSCC migration speed decreased by ~40% (n = 3 experiments, p<0.0001) when plated in the presence of similar amounts of Dis*Ba*-01. This effect was more pronounced for migration directionality (Fig 1B, S2 Movie). While OSCC plated only in fibronectin exhibited a fast, individual and directionally persistent migration behavior, cells treated with 1μM of Dis*Ba*-01 in the media or in the substrate lost their directionality and moved in circles with a tendency to migrate in pairs (collectively). This selective effect of Dis*Ba*-01 in the tumor cells migration may reflect a differential effect on cell-ECM adhesive properties.

### Dis*Ba*-01 modulates cells adhesive properties of OSCC

**Fig 2:**
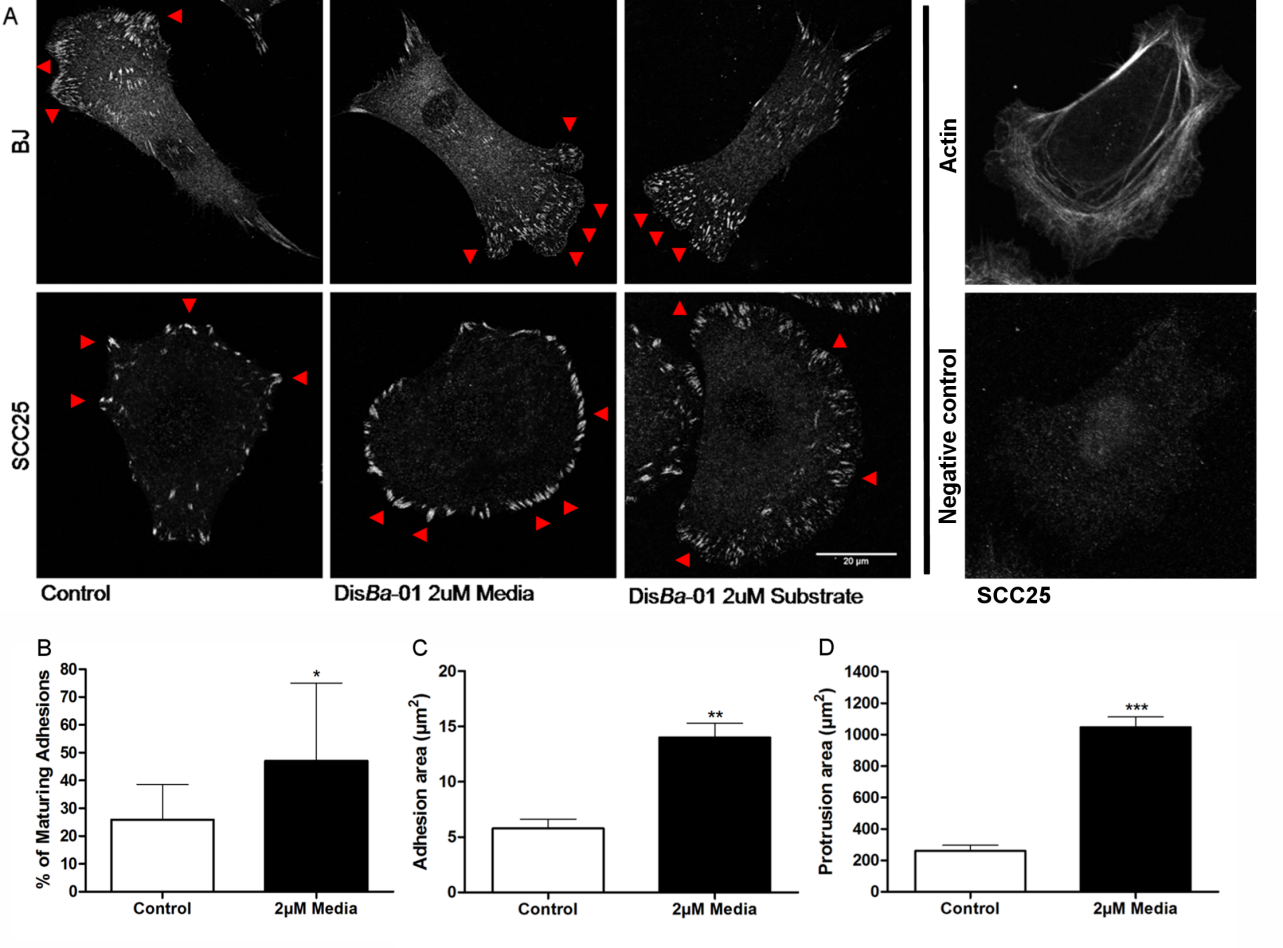
OSCCs treated with Dis*Ba*-01 show an increase in adhesions area and turnover. Cells were allowed to spread for 3 hours on fibronectin (2μg/ml) coated dishes, subsequently fixed, stained for paxillin and analyzed by confocal microscopy. **(**A) Fibroblasts show a large number of small adhesions (red arrow) with no differences between cells with or without Dis*Ba*-01 treatment, while SSC25 treated cells show larger (red arrow) adhesions and rounded shape morphology in the presence of Dis*Ba*-01 (2μM). Actin staining and negative control are shown on the right. (B) Percentage of maturing adhesions on TIRF time-lapse movies in the absence or presence of Dis*Ba*-01, (C) in the adhesion area (D) and in the protrusions area (p<0.05). Data was obtained from 3 independent experiments resulting in the analysis of 13–15 protrusions and 64–84 adhesions per experimental condition.

We studied whether Dis*Ba*-01effects arose from altered adhesion by immunostaining OSCCs and fibroblasts for paxillin, a marker for nascent adhesions [31].Fibroblasts have small adhesions at the cell border, which are present in rapidly migrating but not stationary cells. Dis*Ba*-01 showed no alterations on paxillin distribution for these cells (Fig 2A). In contrast, significant changes were observed in the adhesions in OSCCs, including an increase in the size and number of adhesions in the presence of Dis*Ba*-01 in the media or in the substrate when compared to cells plated only on fibronectin (Fig 2B).

To further investigate the adhesion dynamics seen at the paxillin immunostaining, SCC25 cells were transfected with paxillin-GFP and plated on fibronectin coated dishes (2μg/mL) 24h later in the presence/absence of Dis*Ba*-01 in the media or in the substrate. Then, maturation rate was imaged on TIRF microscopy every 3 seconds during 20 minutes. Dis*Ba*-01 increased the rate of adhesion maturation and the average area of each adhesion in OSCCs (Fig 2, S3 Movie). During the migration process, cells extend their membrane to form the lamellipodia and then attach the membrane to the extracellular matrix. The area of these protruding membranes was also measured and it was ~3x larger in Dis*Ba*-01 treated OSCC than control cells (n = 3 cells, p>0.0005). Taken together, these data show that Dis*Ba*-01exhibits a differential cell type dependent effect on cell adhesion properties.

**Fig 3:**
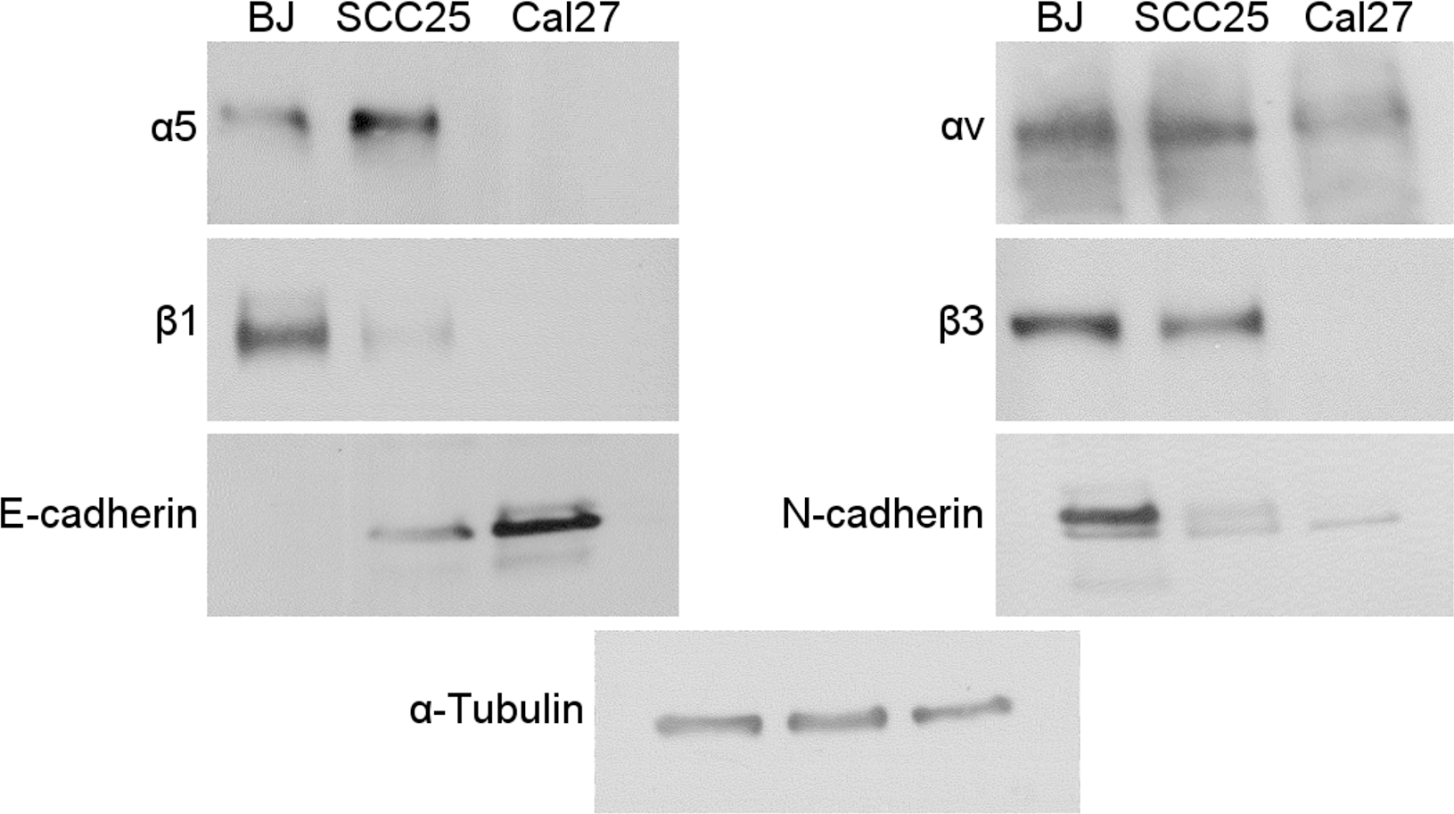
Fibronectin-related integrin receptors show differential expression according to the differentiation level of the tumor cell. Cells lysates of fibroblasts (BJ), highly invasive OSCC (SCC25) or poor invasive OSCC (Cal27 were submitted to western blot for analysis of fibronectin-related integrins. BJ and SCC25 contain α_v_, β_3_ and α_5_ integrin subunits in similar amounts while β_1_ is present in smaller amounts on SCC25 cells. E-cadherin and N-cadherin are differentiation markers.

Fibronectin is a glycoprotein found on the ECM that interacts mainly with integrins α_v_β_3_ and α_5_β_1_ through the RGD motifs in its structure [15, 32]. A possible mechanism for the differential effects of Dis*Ba*-01 on adhesion properties is that the cell lines show different levels of integrin receptors for fibronectin. To test this hypothesis, we analyzed the integrin expression level on fibroblasts (BJ), a highly invasive/low differentiated (SCC25) and a low invasive/highly differentiated (Cal27) OSCC cell line (Fig 3). We observed that the low invasive/high differentiated OSCC cell line (Cal27) presents only low levels of αv subunit, which might explain the poor migratory performance observed on fibronectin [8]. However, fibroblasts showed high levels of all fibronectin-related integrin subunits, which corroborates to the fast single cell migration phenotype. When we analyzed the integrin levels of the highly invasive OSCC cell line, besides the high level of alpha-subunits, these cells show low levels of β1 and high levels of β3 subunits, which indicates that the migration process of this epithelial-derived tumor cell line on fibronectin relies mostly on β3 activity. Since Dis*Ba*-01 is an RGD disintegrin, this result suggests that Dis*Ba*-01 could be selectively acting on α_v_β_3_ integrin of OSCC cells. Since highly invasive OSCC cells migrate in a collective way in matrigel but individually in fibronectin [8], we also tested if Dis*Ba*-01 could inhibit matrigel invasion. In fact, Dis*Ba*-01 (1 and 2 μM) inhibited OSCC invasion (approx. 40 and 50%, respectively) through matrigel (S1 Fig), thus indicating that this disintegrin can inhibit both forms of migration.

**Fig 4:**
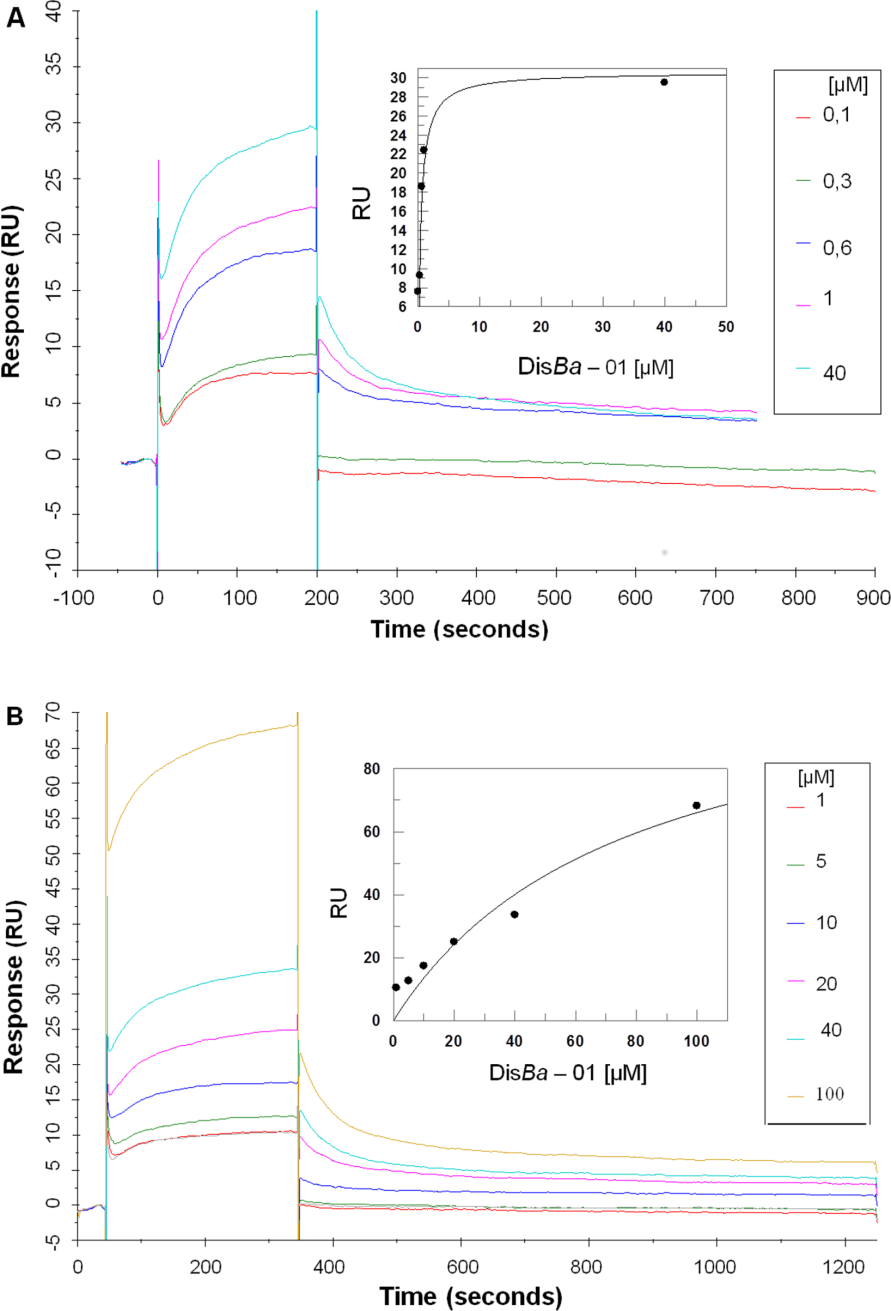
DisBa-01 interacts specifically with integrin α_V_β_3._ (A) Response of increasing concentrations of Dis*Ba*-01 interacting with the α_V_β_3_ integrin immobilized to the sensor chip. Starting at the time “0” and increasing its affinity as DisBa-01 concentration increases. (B) Response of increasing concentrations of Dis*Ba*-01 interacting with the α_5_β_1_. As the concentration of Dis*Ba*-01 increases, the affinity curves show very little displacement. (Single column- print in color)

To further analyze this result and confirm that the effects of Dis*Ba*-01 on migration and adhesion of OSCC cells were caused by the interaction of the disintegrin with the α_v_β_3_ and not α_5_β_1_integrin, the main receptors involved in fibronectin binding [15], the binding between the disintegrin and purified integrins (α_v_β_3_, α_5_β_1_) were analyzed by SPR. The interaction between Dis*Ba*-01 and the α_v_β_3_ integrin was about 100x higher than that for α_5_β_1_integrin (*K*_d_ 7.62 x10^–5^M) indicating a stronger affinity of the disintegrin for the α_v_β_3_integrin. This difference can also be seen in Fig 4.

### β3 overexpression recovered Dis*Ba*-01 effects

**Fig 5:**
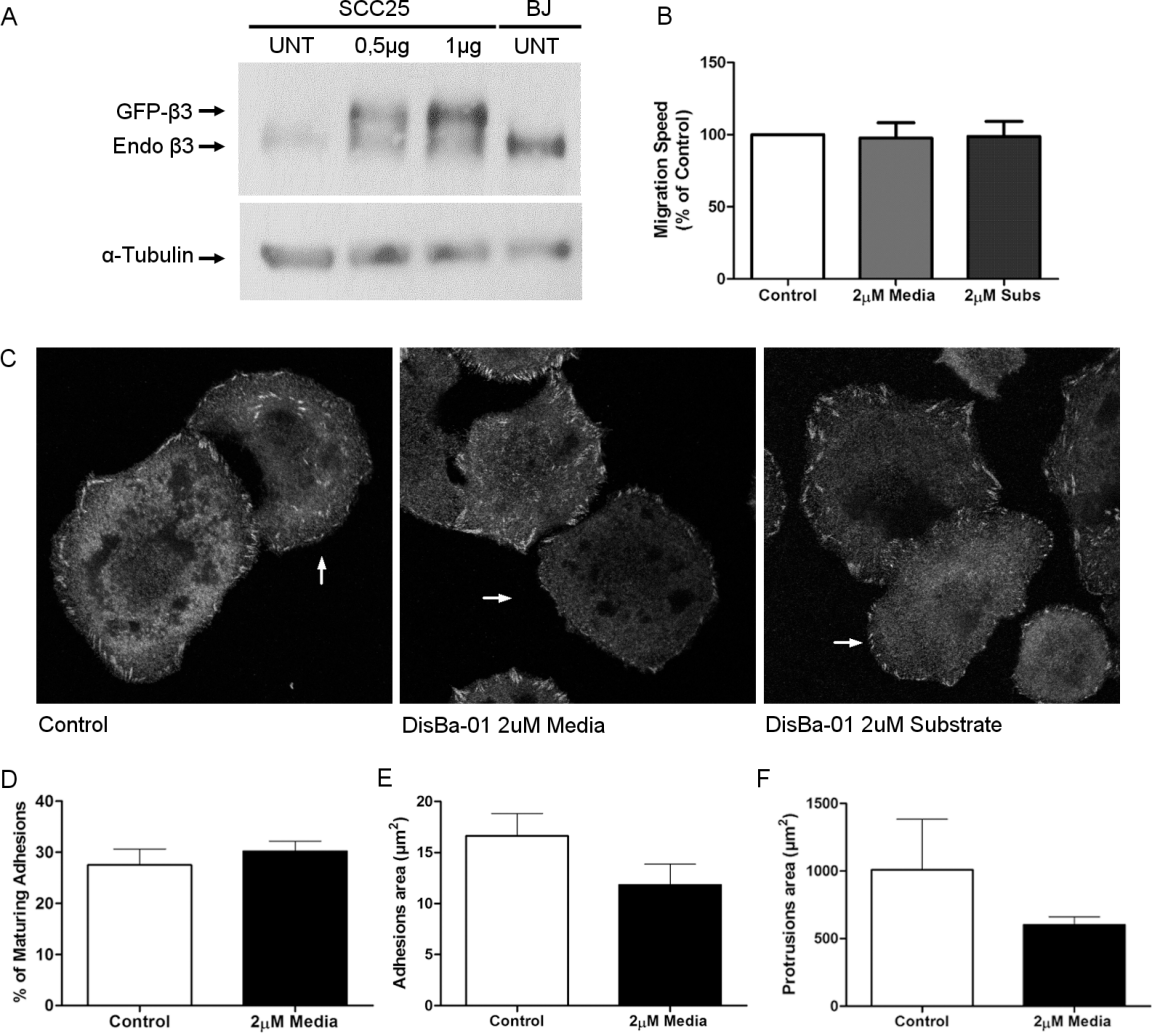
Overexpression of β3 subunit recovered DisBa-01 effects on migration speed and adhesion dynamics of OSCC cells. (A) SCC25 cells were transfected with GFP-β_3_ 0.5 and 1μg of plasmid. Protein bands show the levels of GFP-β_3_ and endogenous β_3_ expressed by SCC25 cells. (B) Migrations speed analysis shows no differences between control and Dis*Ba*-01 treated cells. (C) Representative images of β_3_ overexpressing cells (indicated with arrows) stained for paxillin. (D) β_3_ overexpressing cells with Dis*Ba*-01 reduced the number of adhesions maturation, similar to the controls, when compared with the previous TIRF experiment. (E) DisBa-01 effect over the area of adhesions and (F) area of protrusion was also was reverted on β_3_ overexpressing cells treated with Dis*Ba*-01.

Dis*Ba*-01 interacts with α_v_β_3_ integrins [26], suggesting that the interaction between Dis*Ba*-01 and integrins impairs the migratory activity of OSCC cells. To test this hypothesis, OSCCs were transfected with β_3_ subunit, plated on fibronectin-covered dishes in the presence/absence of Dis*Ba*-01, and migration properties were assessed. Expression levels of two plasmid concentrations for β3 subunit had shown that the lower concentration had little effect whereas the higher concentration, the chosen one, was closer to the BJ integrin levels (Fig 5A, S4 Movie). It was observed that the overexpression of β_3_ subunit restored the migration speed and directionality of cells plated in the presence of Dis*Ba*-01. Adhesion and protrusion areas of cells overexpressing β_3_ integrin with or without Dis*Ba*-01 were assessed both by immunostaining for paxillin and co-transfection with paxillin-mkousing confocal and TIRF microscopy, respectively. Overexpression of β_3_ integrin rescued cells from a phenotype of larger adhesions and protrusions and slower migration (Fig 5 and S5 Movie) such as the phenotype induced byDis*Ba*-01 (2μM), which corroborates with the hypothesis that the inhibition of β_3_ integrin by Dis*Ba*-01is the probable mechanism by which Dis*Ba*-01 impairs the migration properties of OSCC cells.

## Discussion

Tumor metastasis is a major cause of clinical failure in cancer treatment and might be influenced by extrinsic factors, such as the composition of the environment, as well as by intrinsic migratory properties of tumor cells. Also, tumor cell plasticity is a response to micro environmental changes and often influences tumor progression, invasion and resistance to therapy [33, 34]. Alterations in matrix density, pliability, and composition, the nature of the adhesion receptors such as integrins or cadherins, respectively, and expression of cytoskeletal adaptor proteins can lead to interconvertible changes on migration mode depending on each cell intrinsic capacities [8, 34]. Therefore, the blockage of the interaction of tumor cells with the extracellular matrix is an interesting alternative for complementary therapies. For instance, cilengitide is a RGD cyclic pentapeptide that selectively inhibits of αvβ5 and α_v_β_3_ integrins and results from phase 2 trials show an antitumor activity against glioblastoma although it failed to improve patient outcome in phase 3 trials [35–36]. Despite this fact, targeting integrins remains an interesting strategy to disrupt cell-matrix interactions in cancer therapy, according to the authors.

We demonstrated that Dis*Ba*-01, an RGD disintegrin, present on media or substrate, was able to significantly decrease cell migration and cell directionality of highly invasive OSCC, on fibronectin coated dishes but it did not affect fibroblasts. Since soluble and immobilized disintegrin forms triggered opposite results in the past [37], both forms were tested. Migration initiates with biochemical or mechanical cues from the extracellular environment [11, 38]. As demonstrated by previous studies, fibronectin induces migration of different cells lines including low and highly differentiated OSCC cells [8, 39–40]. One interesting finding was that OSCC cells in the presence of Dis*Ba*-01 migrated in a similar fashion of low invasive OSCC cells, Cal27, also plated over fibronectin. Cells moved in circles and in a collective way, indicating loss of directionality and a change on the migration mode that leads toward a less aggressive cell migration phenotype [8]. Another small RGD containing motif disintegrin, eristostatin, was reported to decrease migration speed in a wound healing assay using fibronectin coated plates and melanoma cells. Although it is not mentioned on the report, it is possible to notice impairments on cells directionality and changes on cells morphology especially on the highly invasive melanoma cell c8161 time-lapse videos [18]. According to Missirlis et al [39] the inhibition of α_v_β_3_ or α_5_β_1_ integrins, both on fibronectin coated surfaces caused cells to lose their directionality, but with increased migration speed [39]. Cilengitide was also able to disrupt cell migration, in time-lapse assays, decreasing by ~40%the migration speed in glioblastoma-derived cells, although cells were not plated over fibronectin [41].

A possible mechanism of action for the impairment of cell migration by Dis*Ba*-01 is the modulation of the adhesions. In order to better understand our results on the effects of Dis*Ba*-01 on OSCC migration, we looked deeper into cell adhesion dynamics and morphology. Fast migratory cells, such as leucocytes, are characterized by small and rapidly turning over adhesions while slow adherent migratory cell possesses persistent adhesions that mature into larger and elongated focal complexes [11, 40]. Highly invasive OSCC cells have small and dynamic adhesions when plated on fibronectin, as it was already demonstrated [38]. Adhesion and protrusion sizes were measured and when Dis*Ba*-01 was added to the cells, adhesions became larger, with a higher rate of maturation and some instability could be noticed on membrane protrusions. Blocking of α5β1but not α_v_β_3_ integrin by a specific ligand also increased adhesion sizes [39]. So adhesions function as anchors that respond to different stimuli and forces from the ECM and/or cytoskeleton altering its shape, size and dynamics according to the environment during the migration process [42–44].

Variations in integrin expression could lead to different migratory phenotypes and how cells are affected by Dis*Ba*-01. For instance, the blockage of β3 subunit by DisBa-01 did not affect the migration performance of fibroblasts probably due to the high amounts of both β1 and β3 subunits present in this cell. However, our results on the migration speed inhibition of OSCC could be due to Dis*Ba*-01 interaction with α_v_β_3_ integrin that leaves cells with no proper traction forces to migrate, although they are still adherent, since β1 subunit is present in very limited amount. Moreover, β1 subunit has been characterized for promoting cell migration with no directionality on fibronectin coated surfaces, [45] such as the motile behavior seen on highly aggressive OSCC cell line in the presence of Dis*Ba*-01. This hypothesis was confirmed by the higher affinity that Dis*Ba*-01 has for α_v_β_3_ integrin, as Ramos et al [26] first demonstrated and confirmed here, and its lower affinity for the integrin α_5_β_1_. Finally, the overexpression of β3 subunit on OSCC rescued the migratory phenotype once altered by Dis*Ba*-01 supporting the hypothesis that the major receptor of Dis*Ba*-01 is the integrin α_v_β_3_. These data show that, during invasion, epithelial-derived tumor cells switch the expression of integrins, favoring those which interact with the connective tissue, and that the specific blockage of these receptors, might impair the invasive process. On the other hand, silencing of α_v_β_3_ integrin was recently demonstrated to decrease migration of both malignant glioma and triple negative breast cancer cells [46, 47]. These studies are in complete agreement with our results.

To find new therapies for oral cancers, we showed that an RGD disintegrin changes the migration behavior of an aggressive cancer cell by decreasing its migration speed, directionality and changing adhesion phenotype upon α_v_β_3_ integrin binding. This interesting phenomenon highlights cell plasticity and turning it to our own benefit could be an achievable way to fight against invasion and metastasis.

## Supporting information

S1 Fig Inhibition of cell invasion on matrigel in the presence of Dis*Ba*-01. The cells were plated on the matrigel invasion inserts in the presence of Dis*Ba*-01 (1 μM and 2 μM) for 48 h. Invasion was expressed as a percentage of the control (100%). Cells were counted with an automated fluorescence microscope system, ImageXpress Micro (Molecular Devices). (* p<0.05 compared to positive control).
(TIF)

S1 Movie Fibroblasts migration in presence the absence/presence of Dis*Ba*-01 (2μM). Time-lapse images captured during 8 hours at every 10 minutes (left column) and cell tracking of migratory cells (right column). Fibroblasts were plated over fibronectin (upper line), fibronectin + DisBa-01 (2μM) added to the media (center line) and fibronectin + DisBa-01 (2μM) coated plates (bottom line).
(AVI)

S2 Movie Highly invasive cells (OSCC) migration in presence the absence/presence of Dis*Ba*-01 (2μM). Time-lapse images captured during 8 hours at every 10 minutes (left column) and cell tracking of migratory cells (right column). OSCC were plated over fibronectin (upper line), fibronectin + Dis*Ba*-01 (2μM) added to the media (center line) and fibronectin + Dis*Ba*-01 (2μM) coated plates (bottom line).
(AVI)

S3 Movie Adhesion dynamics of highly invasive OSCC cells. OSCC cells (SCC25) expressing the adhesion marker paxillin tagged with GFP were plated in fibronectin (left) or fibronectin + Dis*Ba*-01 (2μM) in the media and imaged during 20 minutes at every 3 seconds using Total Internal Reflectance Fluorescent (TIRF).
(AVI)

S4 Movie β3 overexpressing highly invasive cells (OSCC) migration in presence the absence/presence of Dis*Ba*-01 (2μM). Overexpressing β3OSCC subunit with images being captured during 8 hours and every 10 minutes (left column) and cell tracking (right column). Overexpressing β3OSCC migrating over fibronectin (upper line), overexpressing β3OSCC migrating over fibronectin with Dis*Ba*-01 (2μM) added to the media (center line) and overexpressing β3OSCC migrating over fibronectin and Dis*Ba*-01 (2μM) coated plates (bottom line).
(AVI)

S5 Movie Adhesion dynamics of highly invasive OSCC cells. OSCC cells (SCC25) expressing the adhesion marker paxillin tagged with mKO and β3 integrin were plated in fibronectin (left) or fibronectin + Dis*Ba*-01 (2μM) in the media and imaged during 20 minutes at every 3 seconds using Total Internal Reflectance Fluorescent (TIRF).
(AVI)
