# Supplementary figures and images for: Inhibition of αvβ3 integrin induces loss of cell directionality of oral squamous carcinoma cells (OSCC)
Source: PLoS One. 2017 Apr 24;12(4):e0176226. doi: 10.1371/journal.pone.0176226 (PMC5402964; doi:10.1371/journal.pone.0176226)

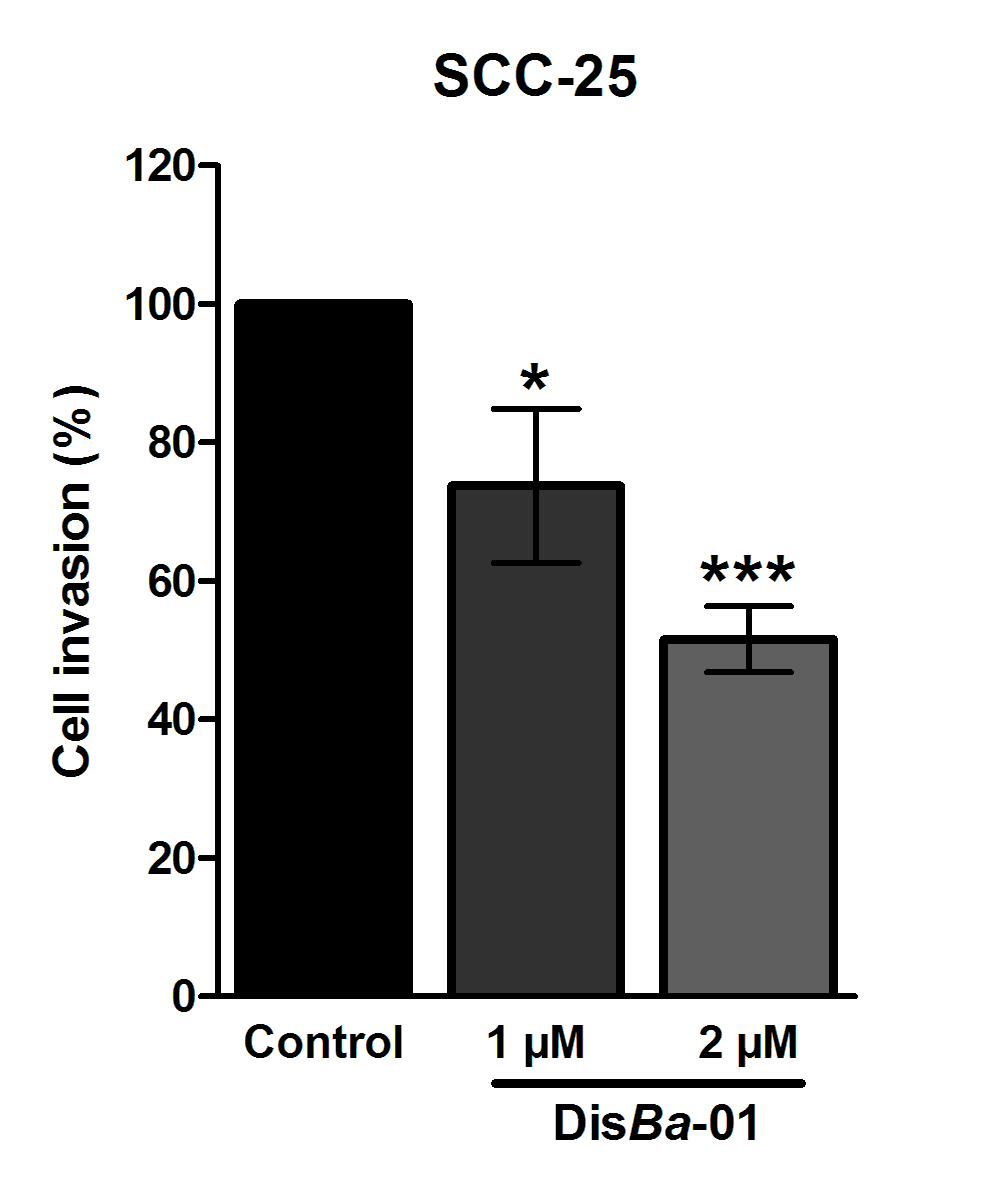

Supplement: S1 Fig — The cells were plated on the matrigel invasion inserts in the presence of DisBa-01 (1 μM and 2 μM) for 48 h. Invasion was expressed as a percentage of the control (100%). Cells were counted with an automated fluorescence microscope system, ImageXpress Micro (Molecular Devices). (* p<0.05 compared to positive control). (TIF) [file pone.0176226.s001.tif]
